# Epigenetics of Homocystinuria, Hydrogen Sulfide, and Circadian Clock Ablation in Cardiovascular–Renal Disease

**DOI:** 10.3390/cimb46120824

**Published:** 2024-12-05

**Authors:** Suresh C. Tyagi

**Affiliations:** Department of Physiology, University of Louisville School of Medicine, Louisville, KY 40202, USA; suresh.tyagi@louisville.edu

**Keywords:** folate 1-carbon metabolism, heart failure, ATP-citrate lyase, gene writer, gene eraser and editor, Piezo, interoception, HFpEF, HFrEF, AKI, CKD

## Abstract

Morning-time heart attacks are associated with an ablation in the sleep-time dip in blood pressure, the mechanism of which is unknown. The epigenetic changes are the hallmark of sleep and circadian clock disruption and homocystinuria (HHcy). The homocystinuria causes ablation in the dip in blood pressure during sleep. Interestingly, HHcy is generated during the epigenetic gene turning off and turning on (i.e., imprinting) by methylation of the DNA promoter. The mitochondrial sulfur metabolism by 3-mercaptopyruvate sulfur transferase (3MST), ATP citrate lyase (ACYL), and epigenetic rhythmic methylation are regulated by folate 1-carbon metabolism (FOCM), i.e., the methionine (M)-SAM-SAH-Hcy, adenosine, and uric acid cycle. Epigenetic gene writer (DNMT), gene eraser (TET/FTO), and editor de-aminase (ADAR) regulate the rhythmic, i.e., reversible methylation/demethylation of H3K4, H3K9, H4K20, m^6^A, and m^5^C. The mitochondrial ATP citrate cycle and creatine kinase (CK) regulate chromatin transcription, maturation, and accessibility as well as muscle function. The transcription is regulated by methylation. The maturation and accessibility are controlled by acetylation. However, it is unclear whether a high fat dysbiotic diet (HFD) causes dysrhythmic expression of the gene writer, eraser, and editor, creating hyperuricemia and cardiac and renal dysfunction. We hypothesized that an HFD increases the gene writer (DNMT1) and editor (ADAR), decreases the eraser (TET/FTO), and increases uric acid to cause chronic diseases. This increases the levels of H3K4, H3K9, H4K20, m^6^A, and m^5^C. Interestingly, the DNMT1KO mitigates. Further, the DNMT1KO and ADAR inhibition attenuate HFD-induced NGAL/FGF23/TMPRSS2/MMP2, 9, 13, and uric acid levels and improve cardiac and renal remodeling. Although the novel role of nerve endings by the Piezo channels (i.e., the combination of ENaC, VDAC, TRPV, K^+^, and Mg^2+^ channels) in the interoception is suggested, interestingly, we and others have shown mechanisms independent of the nerve, by interoception, such as the cargo of the exosome in denervation models of heart failure. If proper and appropriate levels of these enzymes are available to covert homocysteine to hydrogen sulfide (H_2_S) during homocystinuria, then the H_2_S can potentially serve as a newer form of treatment for morning heart attacks and renal sulfur transsulfuration transport diseases.

## 1. Introduction

Combat soldiers, shift workers, and anxiety/depression patients face the formidable consequences of sleep deprivation and cardiovascular and renal diseases. The role of disruption in sleep and the circadian clock in chronic diseases is suggested [1,2,3,4,5,6,7,8,9,10]; however, the mechanism(s) are far from being understood. We discuss a paradigm shift mechanistic pathway that causes the circadian clock-related diseases through the activation of the superior cervical ganglion (SCG) and the excitatory neurotransmitter receptor NMDA-R1 by disrupted epigenetic folate 1-carbon metabolism (FOCM) and increased homocysteine (Hcy, i.e., homocystinuria, HHcy) [11,12,13,14]. In addition, although the inhibition of ATP citrate lyase (ACLYi) attenuates hyperlipidemia [15,16], and ACLY acetylates the histones by epigenetic mechanisms [17], their role in heart failure and the epigenetic control of homocystinuria is unknown. Here we suggest that ACYL inhibition mitigates heart failure and renal homocystinuria by FOCM.

## 2. Discussion

Studies have demonstrated the overactivation of the sympathetic superior cervical ganglion (SCG) by homocystinuria [18] and the disruption of the sleep–wake pattern [19,20,21]. Interestingly, sympathetic denervation has been identified as an underlying cause of the activation of the excitatory neurotransmitter receptor (NMDA-R1) in heart disease. The cardiac neuronal, interstitial, and perivascular fibrosis and ECM remodeling are the reasons for overactivation of the superior cervical ganglia and cardiac hypertrophy during clock ablation-induced heart failure [22]. The severe damage to the endocardial and coronary endothelia by chronic stresses causes overactivation of the cardiac excitatory neurotransmitter (NMDA-R1) and suppression of the cardiac inhibitory neurotransmitter (GABA-R1), suggesting dysregulation of sympathetic and parasympathetic control of the heart, i.e., paradoxical contraction to acetylcholine [23,24]. Interestingly, the association of HFpEF with extra-cardiac features [25], including disorders of the central and peripheral clock system, has been suggested [26,27]. The mechanisms are unclear. The denervation of the heart mitigated the pacing-induced heart failure [28], suggesting a role of sympathetic activation contributing to heart failure [29]. Interestingly, studies have also suggested a relationship between NMDAergic (sympathetic) and GABAergic (parasympathetic) to BMAL1 and Period (Per 2) [30,31,32,33,34], contributing to chronic neuronal overactivation and neuro-inflammation during clock ablation-induced heart failure.

**Figure 1:**
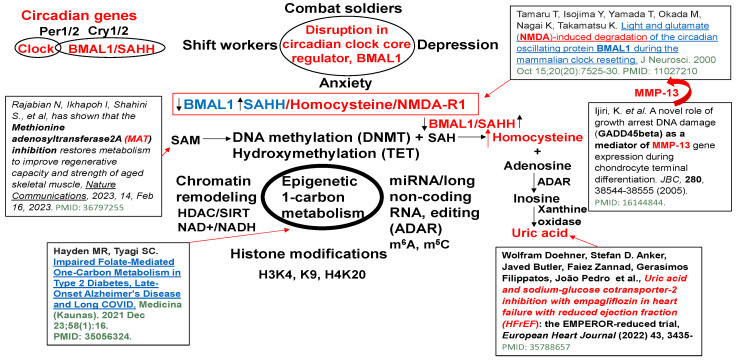
Dysregulation in epigenetic 1-carbon metabolism and Hcy generation causes the disruption of the circadian clock gene core regulator, BMAL1, associated with SAHH/Hcy generation, activating NMDA-R1, and causing anxiety/depression and sleep disturbance [31,37,38,39,40].

**Figure 2:**
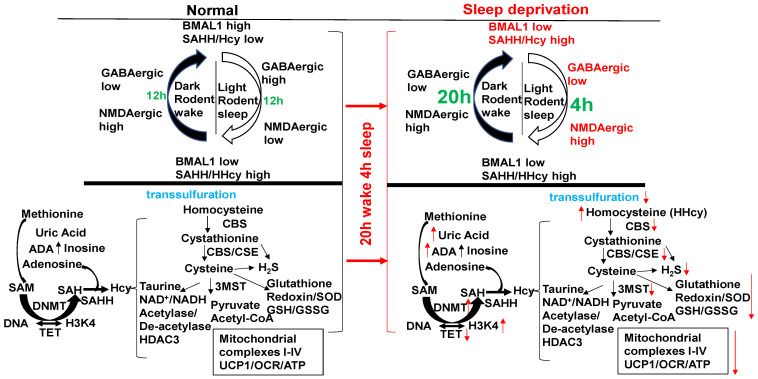
The circadian clock disruption in the sleep/wake cycle (20 h wake/4 h sleep) via BMAL1/SAHH induces epigenetic gene writer (DNMT) and decreases in gene eraser (TET), creating HHcy, activating NMDAergic, leading to HFrEF. Melatonin (NMDAR1 antagonist) mitigates HFrEF. Unlike diurnal humans, mice sleep in the day/light and wake in the night/dark (nocturnal). With a “single hit”, aorta–vena cava fistula (AVF) without injury, the heart creates HFpEF in 6–8 wks through the activity of MMP1/ADAMTS1.

S-adenosyl homocysteine hydrolase (SAHH) controls the circadian clock gene transcription by interacting with the core clock regulator BMAL1 (Figure 1). The BMAL1–SAHH association occurs at the chromatin, promoting rhythmic H3K4 trimethylation (H3K4me3) and cyclic BMAL1 recruitment of target genes [35]. The basic mechanisms of regulation of circadian genes by the CLOCK-BMAL1 nuclear transcription factor, as well as the downstream Period (Per) and cryptochrome (Cry) genes and clock-controlled genes (CCGs), are important [36]; however, the mechanism is unclear. The trimethylates histone H3K4, on the nucleosomes near the circadian gene, promotes rhythmic methylation and generates homocysteine (Hcy, i.e., homocystinuria, HHcy). The HHcy is a product of the epigenetic folate 1-carbon metabolism (FOCM) cycle, unequivocally (Figure 2). Interestingly, the abrogation in the sleep-time dip in blood pressure is one of the causes of morning heart attacks and homocystinuria. The homocystinuria abrogates the sleep-time dip in blood pressure [19,20,21].

The relationship between the circadian rhythms of genes and the epigenetic regulation of these genes and clock relation are characterized by the specific profile of DNA methylation in CpG-islands, which are associated with the senescence of somatic cells and stem cells [41,42]. It has been shown that circadian rhythms operate by very finely tuned regulation of transcription and are controlled by various epigenetic mechanisms, including the activation of enhancers/suppressors, acetylation/deacetylation of histones and other proteins, as well as DNA methylation [43]. Almost 20% of all genes expressed by the cell are affected by the oscillations associated with circadian rhythms [44,45,46,47]. Circadian regulators control several genes that activate the cell cycle and regulate histone modification, accessibility, maturation, and DNA methylation. Therefore, the approaches for determining the epigenetic age from methylation profiles across CpG islands in individual cells are significant.

Hcy is constitutively generated by SAHH, epigenetic gene regulation by the writer (DNMT) and eraser (TET) [48]. During off-printing and on-printing of the genes by active DNA methylation, Hcy is recycled back to methionine and vice versa by the FOCM pathway [48,49]. However, during dysbiosis and the passive DNA methylation due to TET dysfunction, and an increase in SAHH levels, the Hcy is accumulated (i.e., HHcy) [48] and inhibits the active gene expression. Also, H_2_S (hydrogen sulfide, an antioxidant and a potent neurotransmitter) induces TET2 during HHcy [49]. This reveals a direct link between the clock gene, BMAL1, and homocystinuria in de-activating the normal circadian cycle [35]. This elicits that homocystinuria not only causes developmental dysregulation of gene off-printing and on-printing (i.e., neuro-tube defects) but also causes circadian clock dysregulation, including vascular dementia, spasms, and arrhythmia [13,50].

**Figure 3:**
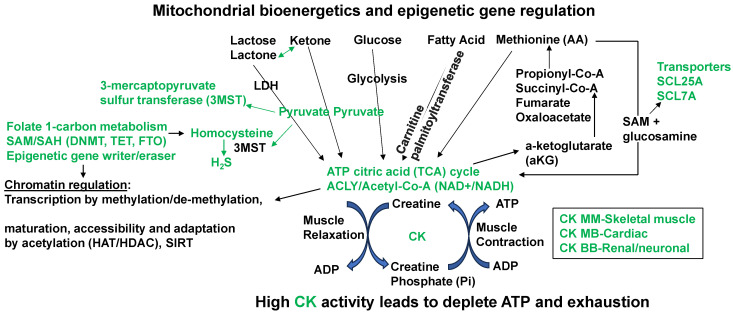
Prominent mitochondrial bioenergetics and epigenetics gene relation biochemical pathways that convert the homocysteine to hydrogen sulfide (H_2_S). The amino acid (AA) methionine modulation of the SAM/SAH ratio in muscle contraction/relaxation and exhaustion of ATP during heart failure. The epigenetic writer (DNMT1) and eraser (TET) via SAM/SAH pathways generate Hcy that is converted to H_2_S by mitochondrial 3MST. The high fat/meat/protein/methionine diet (HFD) decreases muscle contraction by exhausting ATP and increasing creatine kinase (CK) levels.

BMAL1 is a constitutive suppressor of MMP-9 [51]. Conversely, a decrease in BMAL1 levels during wake activates MMPs. Because the growth arrest and DNA-damage-inducible 45 beta (GADD45beta) is an essential mediator of MMP-13 expression during terminal cell differentiation [37], the growth arrest and GADD45beta gene product has been implicated in the stress response, cell cycle arrest, and apoptosis. Therefore, the level of GADD45beta in development and disease is novel. The BMAL1/SAHH/Hcy control of the DMNT/H3K4/TET/GADD45/MMP13 is unique (Figure 1). Homocystinuria exacerbates vasospasm and arrhythmia [13,50,52,53], and these events are mostly affected by clock gene dysregulation. The chromatin transcription is regulated by methylation. The maturation and accessibility are controlled by acetylation (Figure 3). Hcy antagonizes the inhibitory (GABAergic) and agonizes the excitatory (NMDAergic) transmitters [52,54]. Interestingly, muscimol and baclofen are used as GABAergic agonists in the mitigation of anxiety and vascular-associated dementias [55,56,57,58,59,60,61,62,63,64,65,66,67]. Although both melatonin and MK801 are used as NMDAergic blockers/antagonists [68,69,70,71,72,73], their use to mitigate disruptions in the circadian clock system is unknown. Melatonin is a clinically proven antagonist of NMDA-R; therefore, it is significant to use melatonin to mitigate NMDAergic-associated disruption in the circadian clock system [74].

An increase in blood homocysteine, i.e., homocystinuria (HHcy), is also a comorbid condition for clock-mediated cardiovascular diseases. HHcy appears to be associated not only with chronic heart failure but also with acute myocardial infarction (MI) [75,76,77]. There are five ways by which Hcy is accumulated in the plasma and tissues: (i) by a methionine-rich protein diet; (ii) by hyper-demethylation of methionine by methyltransferase (MT) during DNA/RNA methylation reactions; (iii) by hypo-remethylation of Hcy to methionine by MTHFR/vitamin B_12_/folate; (iv) by heterozygous/homozygous mutation in the cystathionine β synthase (CBS), B_6_, and transsulfuration deficiency; and (v) by renal metabolic disease and volume retention.

**Figure 4:**
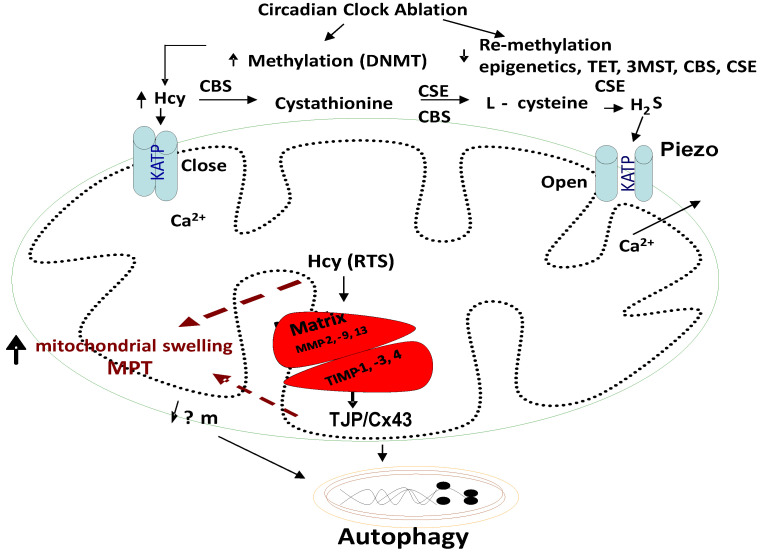
Homocysteine (Hcy) closes Piezo K_ATP_ channels, and H_2_S opens these Piezo channels. Further, the closed K_ATP_ channels trap the calcium ions in the mitochondria and result in arrhythmias and cell death/autophagy; however, the H_2_S reverses these alterations.

Mammalian vascular cells lack the CBS enzyme [78,79]. Many studies have shown that mitochondria play a crucial role in cell survival during ischemia or ischemia–reperfusion (I/R) injury [80]. The I/R injury leads to excessive cytosolic Ca^2+^, mitochondrial Ca^2+^ overload, and a rapid increase in the overall levels of the reactive oxygen species (ROS). It is thought that mitochondrial autophagy, or mitophagy, is the major route by which mitochondria are degraded [7]. Mitochondrial Ca^2+^ (Ca^2+^_m_) overload and oxidative stress are the major triggers of the mitochondrial permeability transition (MPT) and loss of mitochondrial membrane potential (ΔΨm). Further, the mitochondrial permeability transition pore plays an important role in mitophagy [81,82,83]. The mitophagy may play an essential role in maintaining mitochondrial function and genetic integrity (Figure 4).

**Homocysteine, Mitophagy, Ca^2+^ ion, Mitochondrial K_ATP_ Channels, and Hydrogen Sulfide (H_2_S):** Although the novel role of nerve endings in the Piezo channels (i.e., combination of ENaC, TRPV, K^+^, and Mg^2+^ channels) in the interoception is suggested [84,85,86,87,88,89], we and others have shown mechanisms independent of the nerve by interoception, such as the cargo of exosome in denervation models of heart failure [90,91,92,93,94]. The mitochondria also have an ATP-sensitive ‘K^+^’ conductance, recognized as the mitochondrial K_ATP_ (mtK_ATP_) channel [95,96,97,98]. We demonstrated that HHcy decreases myocyte contractile amplitude with the increase in calcium concentration and mitochondrial oxidative stress [77]. An increase in Ca^2+^ influx and oxidative stress in mitochondria leads to mtK_ATP_ channel closing [99]. K_ATP_ channels, when open, shorten the action potential duration and limit Ca^2+^ influx into the myocytes. Activation of mtK_ATP_ by potassium (K^+^) channel openers has been associated with increased survival of cardiac cells following ischemia and improved post-ischemic recovery of heart muscle [100,101]. H_2_S is generated endogenously as a product of the de-sulfuration (i.e., transsulfuration) pathways; however, in the past it has long been labeled a pungent cytotoxic gas, but now it is regarded as the third most endogenous produced signaling gasotransmitter molecule. Furthermore, CBS is the major enzyme that is involved in H_2_S production in the body, but its expression is confined to the brain, kidney, lung and is surprisingly absent in the cardiac tissue [102,103]. The other enzymes that play a role in H_2_S production are cystathionine gamma lyase (CSE) and 3-mercaptopyruvate sulfur transferase (3MST), which produce H_2_S via homocysteine to cysteine metabolism (Figure 1, Figure 2 and Figure 3). The H_2_S has been shown to protect the myocardium from oxidative and endoplasmic reticulum (ER) stress induced by homocystinuria [104,105]. In vivo studies have also demonstrated the efficacy of H_2_S in attenuating myocardial reperfusion injury by protecting mitochondrial function [80,106,107,108,109,110]. Our group has shown that H_2_S protects the cells from oxidative stress induced by homocystinuria [111]. H_2_S levels in human plasma are reported to be ~50 μM, and in vitro studies suggest that it behaves as a vasodilator, and transiently reduces blood pressure by opening K_ATP_ channels [112]. However, the physiological roles of Hcy and H_2_S in mitophagy are not well defined. We speculate that during chronic stress/load, the levels of Hcy are increased and cause mitochondrial calcium mishandling, in part, by closing the mtK_ATP_ channels via mitochondria dysfunction, leading to abnormal mitophagy (Figure 3 and Figure 4).

**Hyperhomocysteinemia (HHcy), Oxidative Stress, Extracellular and Intracellular Matrix Metalloproteinase, Tissue Inhibitors of Metalloproteinases and Mitophagy**: Matrix metalloproteinases (MMPs) and membrane-bound, zinc-dependent endoproteinases are known as collagenases (MMPs-1, -8, and 13), stromelysins (MMP-3 and 10), matrilysins (MMP-7 and -26), membrane-type MMPs (MT-MMPs, MMP-1 to MMP-8), and gelatinases (MMP-2 and 9), and the disintegrin metalloproteinase (including the ADAM). They share structural domains but differ in their substrate specificities [113,114,115,116,117,118,119]. We have shown that the basement membrane matrix of the endothelium mostly contains latent MMPs in part due to the coordination of active-site zinc ions with constitutive nitric oxide in a ternary complex (MMP/NO/TIMP) [120]. Increased oxidative stress leads to generation of nitro-tyrosine residues in the tissue inhibitor of metalloproteinase (TIMP) and release of the active MMP [121]. TIMPs are a family of enzymes that regulate the activity of MMPs, and four have been identified: TIMP-1, -2, -3, and -4 [122,123,124]. Thus, TIMPs play important roles in regulating cellular functions such as invasion, migration, differentiation, and proliferation. These functions are dependent on the cellular matrix composition. During chronic heart failure, increase in the load results in oxidative stress, leading to MMPs activation. We know that oxidative stress plays an important role in the induction of heart failure [125]. Previously, we have found that Hcy induced the generation of ROS production by upregulation of NADPH oxidase and downregulation of thioredoxin in microvascular endothelial cells (MVECs) [120]. Reactive oxygen species (ROS) subsequently induce the synthesis of matrix MMPs in the endothelial cells [126]. We have previously shown that Hcy increases mtROS production, which in turn initiates the mitochondrial membrane depolarization, cytochrome-*c* release, and the activation of caspase-9, thus leading to apoptosis [127]. Recently, several studies have indicated that ROS may be involved in the induction of mitophagy [128,129]. It is suggested that mitochondria are important regulators of apoptosis and mitophagy. Acute activation of MMP-2 leads to a reduction in contractile performance following the ischemia/reperfusion (I/R) injury [130]. We and others have shown the presence of MMPs in the cardiac mitochondria (mtMMP) [131,132,133]. However, the physiological consequence(s) of mtMMPs’ activation is not well understood. Although there is little information regarding the molecular mechanisms by which MMP-2 disrupts the mitochondria, it is well recognized that ROS generated by mitochondria can drive both MMP-2 expression and activation [134]. The activation of MMPs degrades the mitochondrial membrane and impairs mitochondrial function [77,135]. TIMP-1 is induced in heart failure, and TIMP-4 is highly expressed in the heart and is decreased during chronic cardiac failure [136,137,138,139]. TIMP-3 is induced by the loss of mitochondrial membrane potential and the release of cytochrome *c,* which might lead to mitophagy [140,141]. We speculate that in chronic stress/load, the level of Hcy is increased, causing an increase in mitochondrial oxidative stress and activation of latent resident mtMMPs, decreasing the TIMPs, and hence, inducing mitophagy (mitochondrial damage), leading to myocardium dysfunction. We also surmise the mechanism by which activated mtMMPs degrade the mitochondrial membrane and impair the mitochondrial functions, leading to a decline in the contractility of the myocardium.

**Homocystinuria, Mitochondrial Gap Junctions, and the Mitophagy:** Cardiomyocytes are connected cell-to-cell by the intercalated discs, which contain three types of cell junctions: gap, adherens, and desmosomes [113]. Gap junctions (GJs) contain connexin-43. Primarily three connexins are present in the heart. Connexin-37 and -43 are in the endothelium, while connexin-43 and -45 are present in the myocytes [142]. The expression of connexin-43 is reduced in ischemic heart disease [143]. Increasing evidence indicates that connexin-43 interacts with tight junction protein [144]. The downregulation of ZO-1 and claudin-5 expression are matched with the diminished expression levels of connexin-43, suggesting that the tight junction proteins play an important role in the gap junction formation [145,146,147]. Cx43 is abundantly expressed in cardiomyocytes; however, its role in modulating the myocyte mitophagy has not been well established. Cx43 was found to be present in the inner membrane of myocyte mitochondria (mtCx43), and it appears that it is cardioprotective during ischemia/reperfusion injury [148,149,150,151,152,153]. Mitochondrial Cx43 is a novel regulator of mitochondrial functions, and degradation of Cx43 may cause mitophagy [154]. Furthermore, mtCx43 participates in mtK_ATP_-mediated ROS generation and cardioprotection. The hexameric connexin 43 protein forms a large conductance ion channel like the Bcl2 protein. Moreover, Cx channels are voltage gated and can sense mitochondrial membrane potential. The protective role of mtCx43 can be explained based on its interaction with the mitochondrial permeability transition pore, a multiprotein channel that stabilizes the mitochondrial permeability transition pore [154]. Paradoxically, Hcy increases the expression of Cx43 and nitrosylates of Cx43, which causes mitochondrial dysfunction [154,155]. Furthermore, overexpression of Cx43 is associated with the activation of MMPs such as MMP-2 and MMP-9, which increases mitochondrial oxidative stress, activates mtMMPs, and degrades mtCx43, leading to contractile and electrical dysfunction in the cardiomyocytes, in part, by opening mitochondrial permeability transition pores [156]. Mitochondrial Ca^2+^ overload also leads to the opening of the mitochondrial permeability transition pore and the release of inducible factors. The mitochondrial ROS causes the collapse of the mitochondrial membrane potential (ΔΨm), a drop in ATP concentration, a reduction in the cell cycle, and the loss of mtDNA. This suggests that mitophagy in the infarcted heart, in part, leads to the failure of the cellular mitochondrial network and not maintaining ΔΨm, and strongly suggests that mitochondria play a key role in the recovery of electrical activity in the post-ischemic myocardium [157,158] (Figure 4).

**Figure 5:**
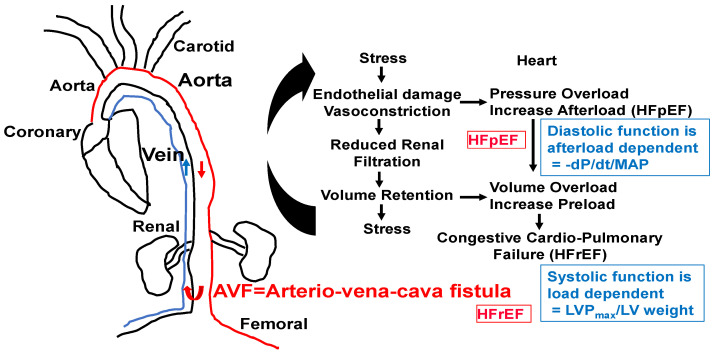
AVF model of congestive HFpEF leading to HFrEF. The hypertrophy and fibrosis are increased with an increase in end-diastolic diameter (EDD), E/e’ ratio, and preserved EF. M-mode short axis ECHO, ventricular filling (E/A ratio), and flow velocity (E/e’ ratio, diastolic function) will be measured. The LVEDD and wall thickness as an index of heart failure will be measured. To determine diastolic dysfunction, LV wall contractile force and LV pressure will be measured by a Millar catheter positioned in the right common carotid artery in anesthetized mice. After measuring aortic pressure, the catheter will be advanced to the LV. Maximum systolic LV pressure (LVP); EDP; and the derivative of fall in pressure after systole, –dP/dt, will be measured. Because diastolic function is afterload-dependent, we will also measure the ratio between the rate of fall in pressure (–dP/dt) and mean arterial pressure (MAP). HFpEF and HFrEF will be distinguished by serial ECHO. HFpEF will be identified by the E/e’ ratio, cardiac fibrosis, and hypertrophy (~6–8 wks post-AVF) and HFrEF by rEF, hypertrophy, and blood–heart barrier (BHB) leakage/LV wall dilatation.

**Figure 6:**
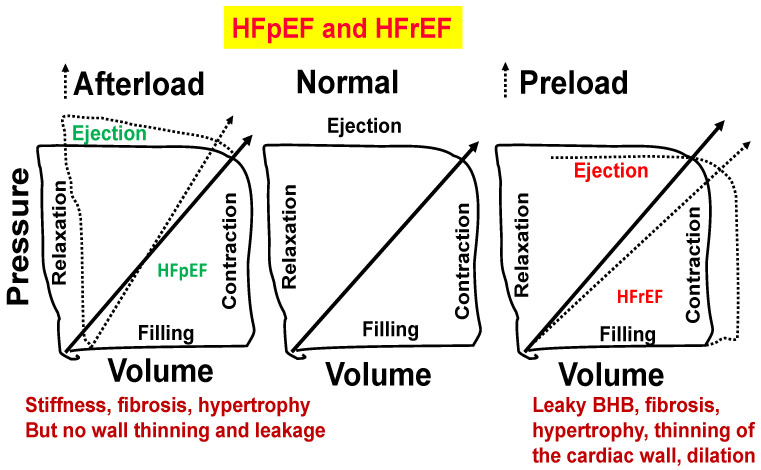
Normal heart (**middle** panel). Stiffness, fibrosis, and hypertrophy but no wall thinning and leakage (**left** panel). Blood–heart barrier (BHB) leakage, myocyte slippage during systole, fibrosis, hypertrophy, thinning of the cardiac wall, and dilation (**right** panel).

Because chronic volume overload increases with age, the increase in venous return by the aorta vena cava fistula (AVF) creates congestive cardiopulmonary heart failure that leads to transition from HFpEF to HFrEF. We suggest that homocysteine antagonizes GABAergic and agonizes NMDAergic [13,14] and contributes to the transition from HFpEF to HFrEF (Figure 5 and Figure 6).

## 3. Conclusions and Future Direction

It is important to investigate circadian rhythm regulation by homocystinuria and its effects on cardiovascular disorders. The circadian periodicity in cardiovascular function and reactivity in relationship to the pathogenesis of cardiovascular disease [38,159,160,161,162], arrhythmia, vascular dementia, and spasm through mechanisms such as epigenetic folate 1-carbon metabolism and DNA methylation by the gene writer and gene eraser are innovative ideas. The idea that inhibitory and excitatory neurotransmitters, respectively, are regulated by the clock gene and epigenetic modifiers during the circadian cycle is novel. The endothelial dysfunction, acetylcholinergic versus muscarinic, and paradoxical vasoconstriction [23] instead of vasodilation, causing vascular dementia, are novel. The hypothesis that the epigenetic dysregulation of clock genes by homocystinuria causes cardiovascular dysfunction, arrhythmia, and vasospasm is novel, and mitigation by an NMDAergic blocker is therapeutically innovative. Homocysteine is metabolized in the body to produce an endogenous gaseous substance, hydrogen sulfide (H_2_S). Despite the experimentally proven protective role of H_2_S in a variety of cardiovascular diseases, the potential role of H_2_S in mitophagy has remained untouched. In this context, we opine that to ameliorate Hcy-induced mitochondrial damage, exogenous H_2_S, with or without folic acid (FA; to lower Hcy levels), should also be investigated. Additionally, the cardioprotective roles of H_2_S and FA need to be investigated. Future research outcome(s) of this novel idea may lead us to better understand the Hcy-induced cardiac remodeling, especially in the beating myocytes. We are confident that such research endeavor will open newer avenues for future investigations regarding the therapeutic potential of this novel gaseous substance in homocystinuria-associated abnormal mitophagy and the associated cardiovascular–renal diseases.
